# Ciprofloxacin-Loaded Polyvinylpyrrolidone Foils for the Topical Treatment of Wound Infections with Methicillin-Resistant *Staphylococcus aureus* (MRSA)

**DOI:** 10.3390/pharmaceutics15071876

**Published:** 2023-07-03

**Authors:** Fiorenza Rancan, Jana Jurisch, Sabrina Hadam, Annika Vogt, Ulrike Blume-Peytavi, Ilker S. Bayer, Marco Contardi, Christoph Schaudinn

**Affiliations:** 1 Department of Dermatology, Venereology and Allergology, Charité—Universitätsmedizin Berlin, Corporate Member of Freie Universität Berlin and Humboldt-Universität zu Berlin, 10117 Berlin, Germanyannika.vogt@charite.de (A.V.); ulrike.blume-peytavi@charite.de (U.B.-P.); 2 Smart Materials, Istituto Italiano di Tecnologia, 16163 Genova, Italy; ilker.bayer@iit.it (I.S.B.); marco.contardi@iit.it (M.C.); 3 Department of Earth and Environmental Sciences (DISAT), University of Milan-Bicocca, Piazza della Scienza, 20126 Milan, Italy; 4 Advanced Light and Electron Microscopy, Zentrum für Biologische Gefahren und Spezielle Pathogene 4, Robert Koch Institute, 13353 Berlin, Germany; schaudinnc@rki.de

**Keywords:** wound dressing, drug delivery, organ culture, infection models, ciprofloxacin, infected wounds

## Abstract

Bacterial infections are a constant challenge in the management of acute and chronic wounds. Chronic wounds, such as diabetic foot ulcers, have increased significantly in the last few years due to the rise of an aging population. A better understanding of the infectious pathophysiological mechanisms is urgently needed along with new options for the treatment of wound infections and wound-healing disorders. New advances in the preparation of biocompatible dressing materials that can be loaded with antimicrobial drugs may improve the topical treatment of infected wounds. In this study, we investigated the antimicrobial activity of polyvinylpyrrolidone (PVP) foils loaded with ciprofloxacin (Cipro-foils) in the presence of acetic acid as a co-solvent. We used ex vivo human wounds that were infected with two bacterial strains: methicillin-resistant *Staphylococcus aureus* (MRSA) or *Pseudomonas aeruginosa* (PAO1). The effectiveness of the treatment was demonstrated by the quantification of the living bacteria extracted from the wound and the detection of released immunological mediators in skin extracts and in the skin culture media. We found that Cipro-foils effectively treated the infection with both PAO1 and MRSA. Other than PAO1, MRSA had no lytic activity toward skin proteins. MRSA infections increased cytokines’ expression and release. Interestingly, treatment with Cipro-foils could partially counteract these effects.

## 1. Introduction

The incidence of infections caused by antibiotic-resistant bacteria has increased over the last decades, and it is more and more recognized as a global health problem to an extent that the name “silent pandemic” has been used [1,2]. Especially, hospital-acquired infections represent a pressure on the healthcare system due to the increased mortality, hospitalization time, and healthcare costs [3,4]. Surgical site infections (SSI) occur in about 2% of all surgeries and make up at least 20% of all nosocomial infections [5]. On the other side, infections can cause considerable complications in the treatment of chronic wounds leading to prolonged and cost-intensive treatments [6,7,8]. The prevalence of all chronic wounds is estimated to be 2.21 per 1000 population, while the prevalence of chronic leg ulcers is 1.51 per 1000 population [9]. Notably, these numbers are going to increase dramatically due to the demographic growth of the elderly population [10]. The annual costs estimation for acute and chronic wound treatments in the USA made by Medicare in 2014 ranged from $28.1 to $96.8 billion [11]. More recently, the annual economic burden of venous leg ulcers associated to deep venous disease in Australia, some Europe countries, and the USA was estimated to be of ~US$10.73 billion in total or US$5527 per person per year [12].

Consequently, new prevention strategies and more affordable treatments against wound infections are urgently needed. Topically applied antiseptics would be of advantage due to the rapid onset of the treatment and high local drug concentrations avoiding systemic toxicity. Importantly, in order to prevent the formation of antibiotic-resistant strains and to reach biofilm-associated bacteria, a sufficiently high concentration of antibiotics should be ensured [13]. Difficulties often lie in the form of application, with liquid formulations drying out and ointments being poorly distributed. The modern treatment of chronic wounds includes wound dressings as a therapeutic principle. Conventional, occlusive wound dressings (e.g., made of gauze) can create an anaerobic wound environment and promote the spread of pathogens. In comparison, hydroactive wound dressings ensure a moist wound environment and an exchange of water and oxygen, which contributes to accelerate the wound healing process. Currently, there are only a few examples of wound dressings with antimicrobial properties. Silver-based materials are the most used local treatments of wound infections. Such dressings can efficiently prevent and treat infections; however, they are expensive and not free from safety and environment-related concerns. In addition, reports on silver-resistant bacteria are increasing [14,15,16]. Novel biomaterials such as nanofibers, hydrogels, and films made of alginate, chitosan, collagen etc., can also be combined with antimicrobial agents or other substances relevant to wound healing and thus ensure the treatment and closure of infected wounds within one application [17,18,19,20,21,22]. Such dressings may represent very attractive alternative options to oral antibiotic therapies. Among the several wound dressings presented in the literature, dressings based on poly-vinylpyrrolidone (PVP) showed promising results in the management of infected and non-healing wounds [23]. This polymer can ensure: (i) a high level of bio- and hemocompatibility, (ii) adhesion properties comparable to commercial patches, (iii) excellent water absorption, and (iv) controllable release of drugs when combined with other polymers [24].

In general, to test the antimicrobial efficacy of newly developed dressing materials, in vivo rodent models are used. However, in addition to the fact that for ethical reasons animal experiments should be kept to a minimum, the translation of such results to humans is limited due to the large anatomical and physiological differences between human and rodent skin. Further models consist of three-dimensional reconstructed skin made of differentiated keratinocytes and fibroblasts. These in vitro models cannot reflect the complex reaction of the skin as an immune organ because of the lack of skin cell populations such as mast cells, dendritic cells, skin-resident T and B cells. In order to circumvent these differences and limitations, a wound infection model was developed based on human skin explants where superficial wounds were created and inoculated with *Pseudomonas aeruginosa* [25]. Such a model was intended to simulate poorly perfused wounds characterized by prolonged or increased inflammatory phase and associated with bacterial infections and biofilm formation. *P. aeruginosa* bacteria could proliferate in the ex vivo wounds and form biofilm-like structures. It was also observed that the PAO1 strain had strong proteolytic effects and that the cutaneous immune response was significantly reduced with respect to untreated controls. In this study, we used an improved protocol, in which after infection, skin was cultured at the air–liquid interface. Furthermore, because methicillin-resistant *Staphylococcus aureus* (MRSA) represents another pathogen commonly isolated from chronic wounds [26] that is particularly threatening in the hospital context, an ex vivo wound model infected with an MRSA strain was also developed and compared to the existing PAO1 wound model. The strains used in this study, PAO1 (ATCC 15692) and MRSA (ATCC 43300), originated from clinical isolates [27,28]. Both strains form biofilms, which lend the bacteria extremely increased resistance to external influences, including disinfectants and antibiotics, and even the ability to withstand the host immune response [29,30].

The two models were used to test foils made of PVP loaded with the antibiotic drug ciprofloxacin (Cipro). Cipro has a low water solubility. The corresponding salt is water soluble but has a different biodistribution. Therefore, Cipro was solubilized using 30% acetic acid and incorporated in the PVP foil. Interestingly, the residual acetic acid (less than 0.1%) conferred to the foil special elasticity properties [31]. The foils dissolve within hours when immersed in water, whereas after application on in vivo or ex vivo wounds, their dissolution depends on the amount of wound exudate. In this study, where skin was culture in a trans-well set-up, we observed that within 20 h after application on the infected wounds, the foils had dissolved to form a gel-like formulation on the top of the wound. The Cipro-foils showed very promising antimicrobial and anti-biofilm properties when tested on the ex vivo PAO1 wound infection model using the established incubation protocol. In this study, we used the new culture protocol and tested the antimicrobial properties of Cipro-foils also with the MRSA infection model. To characterize and compare the two infection models, the distribution of bacteria in the wound tissue was analyzed microscopically using in situ fluorescence hybridization (FISH). The growth of the bacteria and the success of the Cipro-foils treatment was determined by quantifying the living bacteria extracted from wounds. The immunological reaction to the bacteria with and without treatment was analyzed by monitoring the most common inflammatory cytokines.

## 2. Materials and Methods

### 2.1. Preparation and Characterization of PVP Foils Loaded with Ciprofloxacin (Cipro-Foils)

PVP transparent foils were prepared by a solvent-casting method starting from aqueous solutions of PVP (3% *w/v*) with a molecular weight (MW) of 360,000 g/mol (Merck, Milan, Italy), ciprofloxacin monohydrochloride monohydrate (Merck, Milan, Italy, 98.0% HPLC tested purity) and acetic acid (≥99.7%, Merck, Milan, Italy). The detailed preparation protocol is described elsewhere [31]. First, 60 mg of ciprofloxacin was dissolved in acetic acid 30% (*v/v*) and added to the PVP solution to reach a final volume of 30 mL and a final drug concentration of 88 mmol and 5% (*v/v*) of acetic acid. These solutions were poured on Petri dishes (diameter 8.75 cm) and placed under an aspiration hood at room temperature (20 °C and 40–50% relative humidity) for 3 days and successively in a vacuum desiccator for further 3 days.

Infrared spectra of Cipro-foil, PVP/Acetic acid foil, and PVP/Acetic acid/Cipro-foil were acquired by utilizing an ATR accessory (MIRacle ATR, PIKE Technologies, Fitchburg, WI, USA) with a diamond crystal coupled to an FTIR spectrometer (Vertex 70v FTIR, Bruker, Billerica, MA, USA). All spectra were acquired between 4000 and 600 cm^−1^, with a resolution of 4 cm^−1^, accumulating 64 scans. The UV-Visible spectra of Cipro-foil in acid (pH = 4.5) aqueous solutions and of Cipro released from Cipro-foils were analyzed using a CARY 300 Scan UV–visible spectrophotometer (Aligent Technology, Milan, Italy).

### 2.2. Ex Vivo Wound Infection Model and Treatment with Cipro-Foils

The skin material was obtained, after signed consent, from healthy individuals who underwent a plastic surgical procedure. The investigations were carried out with the approval of the ethics committee Charité—Universitätsmedizin Berlin (application no. EA1/135/06, renewed in January 2018) of the Charité University Medicine Berlin, which is in accordance with the ethical principles and guidelines of the Declaration of Helsinki. The excised human skin was collected from the clinic on the day of the surgery and processed within 2–4 h. Skin samples were first roughly cleaned of blood residues and other impurities using phosphate-buffered saline solution (PBS) and then cut into pieces of about 1.5 × 1.5 cm^2^. Excess subcutaneous fat was removed using scissors. Skin pieces were stretched slightly and fixed with cannulas to Styrofoam blocks wrapped in aluminum foil and covered with parafilm (Bemis Company, Neenah, WI, USA). A further cleaning step with PBS and ethanol (70%) followed. To create the wounds, a spherical ball-shaped milling cutter, 6 mm in size (No. 28725, Proxxon, Föhren, Germany), rotating at 16,000 rpm, and mounted on a micro-motor handpiece (Marathon N7, TPC Advanced Technology, Inc., Diamond Bar, CA, USA) was used. In this way, reproducible superficial wounds with a diameter of approximately 5 mm were created. For the infection, PAO1 (ATCC 15692) and MRSA (ATCC 43300) strains were used. The pathogens were thawed from the cryo-stock and cultured overnight in tryptic soy broth (TSB, Corning, Kaiserslautern, Germany) at 37 °C and 150 rpm. The steady-state bacterial suspension had a final concentration of approximately 1 × 10^9^ colony-forming units (CFU)/mL. Starting from this concentration, two dilutions (1:10) with TSB resulted in a suspension of 1 × 10^7^ CFU/mL. Using a 10 μL syringe (26 gauge) with a tapered tip (#002000, SGE Analytical Science, Ringwood Victoria, Australia), 10 µL of the bacterial suspension was taken and injected in the dermis from the wound edge toward the wound center at an angle of about 10°, resulting in an inoculum of ~1 × 10^5^ bacteria per wound. The skin samples were placed in a humid chamber (a box with wet towels and closed with a lid) and incubated at 37 °C, 5% CO_2_, and 95% humidity. In each experiment, skin samples with uninfected wounds were used as a control. After 20 h, skin samples were transferred to 6-well plates each containing 2 mL 1640 RPMI medium with 10% fetal calf serum (FCS). Medium with streptomycin (100 μg/mL, Gibco, Darmstadt, Germany) and penicillin (100 I.E./mL, Merck, Hamburg, Germany) was used for the uninfected controls. For the treatment groups, discs with a diameter of 8 mm were cut from the Cipro-foils using a biopsy punch device and placed on the top of each wound. This resulted in a final concentration of approximately 500 µg antibiotic per wound. The incubation was carried out for a further 20 h in a humidified box placed in an incubator (37 °C, 5% CO_2_, 95% relative humidity). After the incubation, culture media were collected, and the treated wound tissue was removed using a biopsy punch (8 mm). The skin was processed for bacterial count and cytokine measurements. Skin samples were also prepared for cryosections and in situ hybridization to visualize the microorganisms in the wounds.

### 2.3. Fluorescence In Situ Hybridization (FISH)

FISH was used to visualize the pathogens inoculated in the skin samples. This is a molecular–biological method for the direct and specific detection of nucleic acids. Oligonucleotides (probes) marked with fluorescent dyes are used, which bind to specific ribosomal RNA target sequences. The EUB 338 probe (Integrated DNA Technologies, Coralville, IA, USA), Sequence: 5′-GCT GCC TCC CGT AGG AGT-3′, was used to identify the *Pseudomonas aeruginosa* strain PAO1. This is a cross-strain eubacteria probe, which in this case is labeled at the 5′ end with the fluorescent dye cyanine-3 (Cy3). The detection of the MRSA strain was carried out with a *Staphylococcus aureus*-specific probe (5′-GAA GCA AGC TTC TCG TCC G-3′), which is marked with the dye DY549P1. Both probes bind to the 16S rRNA as a target sequence for detecting the bacteria. Cryosections with a thickness of 10 µm were prepared using a Microm HM 560 Leica microtome (Leica Microsystems, Wetzlar, Germany). The slides were stored at −20 °C until further processing. For the staining, slides were thawed at room temperature, and the chosen sections were delimited with a DAKO pen. Sections were fixed for 10 min at room temperature with 20 µL PFA (4%, Merck, Steinheim, Germany). For the samples inoculated with the Gram-positive MRSA, a permeabilization step with Tween-20 (0.02%) for 30 min at room temperature was carried out. For the hybridization, the probes (500 µg/mL) were diluted to a working concentration of 5 ng/mL using a hybridization buffer (5 M NaCl, 1 M Tris pH = 8, 10% SDS and 30% formamide in ddH_2_O). Hybridization was carried out in an oven at 48 °C for 90 min. Two washing steps followed. Skin sections were observed with a confocal laser scanning microscope (LSM-700, Zeiss, Jena, Germany).

### 2.4. Wound Extraction and Quantification of Bacteria

For the quantification of the bacteria, the individual 8 mm biopsies were transferred to a 1.5 mL tube with 0.2 mL of phosphate buffer (Dulbecco PBS, pH 7.4). Tissue was homogenized for 3 min using a sterile steel pistil at 150 rpm mounted on a digital overhead stirrer (DSL, VELP Scientifica Srl, Usmate, MB, Italy). Thereafter, samples were sonicated for 10 min at 40 kHz and 200 W_eff_ using an ultrasonic bath (BactoSonic1, Bandelin, Berlin, Germany). For the quantification of the bacteria number, 80 µL of the wound tissue homogenate was taken and transferred to a 96-well plate. Dilution series with a dilution factor of 10 (1:10 dilution with PBS) were prepared. The remaining sample extracts were stored at −20 °C for protein extraction and subsequent IL-8 analysis using an ELISA assay. Using a multichannel pipette, 5 µL of the individual sample dilutions was taken from each well, applied to a square tryptic soy agar plate (Axon Lab, Reichenbach, Germany) and incubated at 37 °C overnight. The number of bacteria per wound was then calculated, taking into account the number of dilutions.

### 2.5. Protein Extraction and Measurement

For the protein extraction, samples were thawed on ice, mixed with 200 µL of ice-cold extraction buffer (100 mM Tris-HCl, 150 mM NaCl, 1 mM EDTA, 1 g Triton-X-100 in dH_2_O) and mixed well. Incubation was carried out with constant shaking (700 rpm) for 30 min at 4 °C. The individual samples were then sonicated (70 Hz, 240 W_eff_, 10 min) and centrifuged for 10 min at 450× *g*. The supernatant was stored at −20 °C and used for total protein and cytokine determination by ELISA assay. The total amount of protein was determined by the Pierce 660 nm Protein Assay (Thermo Scientific Inc., Rockford, IL, USA) following the manufacturer’s instructions. Absorbance values were measured with the EnSpire Multimode plate reader (Perkin Elmer, Akron, OH, USA). Bovine serum albumin was used as the standard.

### 2.6. ELISA and Multi-Analyte ELISA

The enzyme-linked immunosorbent assay (ELISA) was used to detect IL-8 in the tissue extracts as well as in the culture medium (Human IL-8 CytoSet, Thermo Fisher Scientific Inc., Rockford, IL, USA). The assay was run according to the manufacturer’s instructions. For the determination of other inflammatory cytokines (IFN-γ, TNF-α, GM-CFS, IL-1α, IL-1β, IL-2, IL-4, IL-6, IL-8, IL-10, IL-12 and IL-17A), the protein extracts from three donors were pooled, diluted 2-fold and analyzed using a Human Inflammatory Cytokines Multi-Analyte ELISArray Kit (Qiagen, Hilden, Germany).

### 2.7. Data Analysis and Statistics

In total, skin from 12 donors was used. Each experiment was repeated at least three times using skin from different donors. Data of CFU and IL-8 measurements are reported as dot plots with arithmetic means and the standard error of the mean (SEM) of at least three experiments run in triplicate. Data of the multi-analyte ELISA are reported as bar diagrams of averages and standard errors of duplicates resulting from wound extracts pooled from three donors (three independent experiments). Calculations and statistical analysis were completed with Excel 2007 (Microsoft, Redmond, WA, USA). One-way ANOVA and two-tailed unpaired Student’s t-tests were used. *p* values were reported in the figures as follows: * *p* < 0.05, ** *p* < 0.01, *** *p* < 0.001. Only *p* ≤ 0.05 was regarded as a significant difference. Graphics were created with Prism GraphPad (GraphPad Software, San Diego, CA, USA).

## 3. Results

### 3.1. Characterization of Cipro-Foils

**Figure 1:**
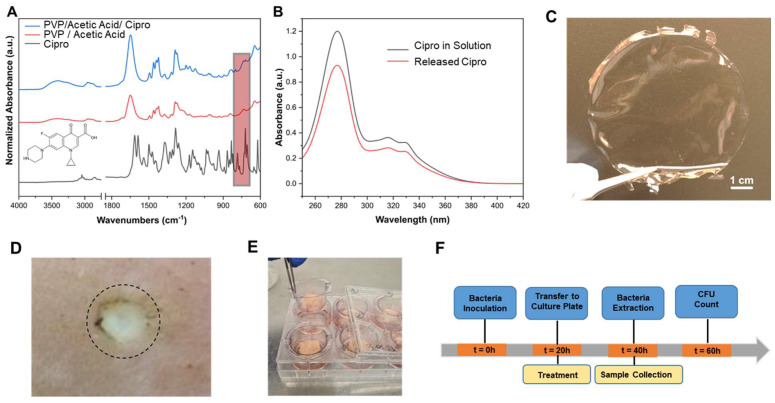
Characterization of the Cipro-foils and schematic illustration of the study. (**A**) FTIR spectra for Cipro, PVP/Acetic acid, and PVP/Acetic acid/Cipro samples. (**B**) UV-Vis spectra for Cipro in solution and Cipro released from the Cipro-foil. (**C**) Picture of a Cipro-foil sample. For the wound treatment, an 8 mm in diameter disc was cut using a punch biopsy and placed on the top of the wound. (**D**) Picture of an ex vivo wound 20 h after infection with PAO1. The circle shows the area treated with the Cipro-foil disc. (**E**) After infection and application of the treatment, skin pieces were transferred to a trans-well insert that was placed on a culture plate with each well filled with 2 mL of RPMI 1640 medium. After 20 h of further incubation samples were collected and the CFU assay was performed. (**F**) Timeline of the experiment.

Cipro-foils were chemically characterized using ATR-FTIR and UV-Vis spectrophotometers. The FTIR spectra of Cipro-foil, PVP/Acetic acid foil, and PVP/Acetic acid/Cipro-foil are reported in Figure 1A. Cipro spectrum presents the typical peaks of the antibiotic: aromatic CH stretching modes at 3016 and 3045 cm^−1^, asymmetric and symmetric stretching of CH and CH_2_ at 2982, 2959, 2912, 2874, and 2843 cm^−1^, C=O stretching modes at 1643 and 1614 cm^−1^, aromatic C=C stretching at 1587 cm^−1^, and CH and CH_2_ rocking modes of Cipro between 735 and 700 cm^−1^. PVP typical peaks were found in the spectrum of PVP/Acetic acid foils: O–H stretching mode at 3403 cm^−1^, asymmetric and symmetric CH_2_ stretching modes at 2988, 2955, 2924, and 2864 cm^−1^, respectively, C=O stretching mode at 1641 cm^−1^, C–N stretching mode at 1018 cm^−1^, out-of-plane C–H bending mode at 844 cm^−1^, and C–H rocking mode at 733 cm^−1^. The PVP/Acetic acid/Cipro spectrum shows the peaks of PVP. Indeed, the peaks of the polymer merge the vibration modes of the antibiotic in the majority of the spectrum. Only the area of the CH and CH_2_ rocking modes of Cipro between 735 and 700 cm^−1^ can be noticed (highlighted by the red square in the Figure 1A), confirming the presence of the drug.

To further investigate the drug stability after the fabrication, UV-Vis spectra of Cipro in an aqueous solution and Cipro released from the Cipro-foil were recorded (Figure 1B). As can be noticed, the two spectra are comparable, confirming that the Cipro-foil was stable and it was not affected by the fabrication procedure.

The obtained Cipro-foils are transparent as shown in Figure 1C. After the inoculation and growth of bacteria in the wound (Figure 1D), the Cipro-foils were applied on the top of the wound and the skin pieces were cultured at the air–liquid interface for a further 20 h (Figure 1E). Wound tissue and culture media were then collected, and CFU were measured. A timeline of the experiment is shown in Figure 1F.

### 3.2. Visualization of Bacteria in the Wound Model (FISH)

**Figure 2:**
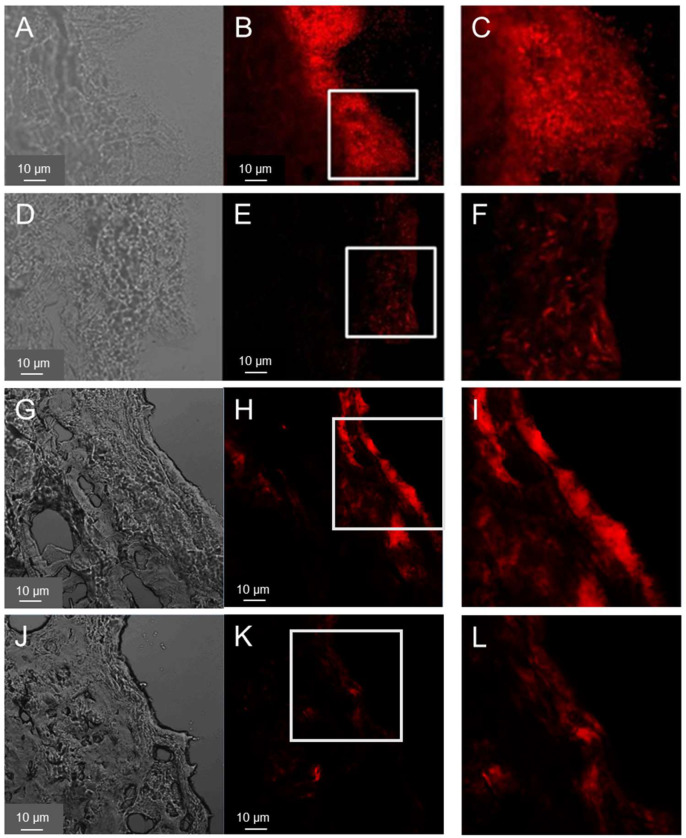
Distribution of PAO1 (**A**–**F**) and MRSA (**G**–**L**) in ex vivo wounds. Bacteria in wound sections were visualized using FISH. Representative images of transmitted (**A**,**D**,**G**,**J**) and fluorescence (**B**,**E**,**H**,**K**) light, taken using confocal laser scanning microscopy and 400× magnification are shown. Magnification (1.5 time) of the inserts is shown in the third column (**C**,**F**,**I**,**L**). Samples 40 h after inoculation (**A**–**C**,**G**–**I**) or 0 h after infection plus 20 h of treatment (**D**–**F**,**J**–**L**) with the Cipro-foils were analyzed.

**Figure 3:**
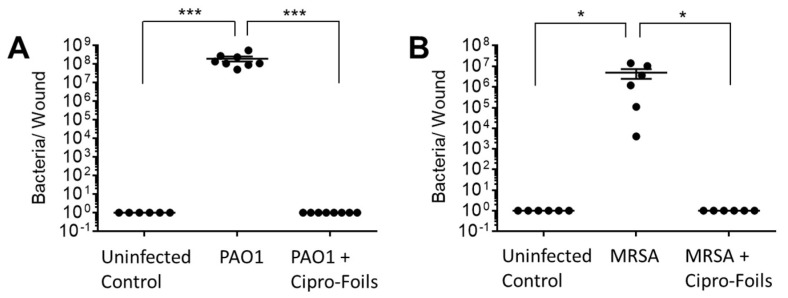
Antimicrobial efficacy of the Cipro-films. CFU count in PAO1 (**A**) and MRSA (**B**) infected wound samples 40 h after and with and without antimicrobial treatment with Cipro-foils (500 µg/wound). Arithmetic average and SEM are shown. * *p* < 0.05; *** *p* < 0.001.

FISH was used to visualize pathogens introduced into the wound tissue. The results are shown in Figure 2A–F for PAO1and Figure 2G–L for MRSA. After 40 h, sections of the wound infected with PAO1 showed clear biofilm-like structures, especially on the surface of the wound bed (Figure 2A–C). PAO1 were also found in the deeper layers of the dermis but far fewer. Similarly, biofilm-like structures could be detected in the samples treated with ciprofloxacin-loaded PVP film but with a weaker fluorescence signal, i.e., much less bacteria (Figure 2D–F). These are likely dead bacteria and suggest that the treatment of the infection was successful as confirmed by the subsequent results of the CFU test (Figure 3).

Cryosections of wounds 40 h after the infection with MRSA showed a distribution of the bacteria on the wound surface but predominately at the wound edges (Figure 2D–I) along with smaller agglomerations in the deeper layers of the wound. In general, the growth of the MRSA biofilm-like structures on the top of the wound was less pronounced. In addition, for the MRSA samples, a reduction in fluorescence intensity (i.e., of the bacteria) was observed after the treatment with the Cipro-foils. These results were confirmed by those of the CFU assay (Figure 3).

### 3.3. Antimicrobial Efficacy of Cipro-Foils

To test the antimicrobial efficacy of the Cipro-foils, tissue extracts were prepared, and the number of living bacteria was assessed using the CFU assay (Figure 3). After 40 h of inoculation without antibiotic treatment, an increase in the number of PAO1 bacteria from the injected 1 × 10^5^ to the extracted 4 × 10^8^ CFU was measured (Figure 3A).

The CFU count from different samples was very reproducible with very low SEM. On the contrary, for the MRSA bacteria, the CFU counted in the extracts differed strongly from sample to sample. On average, 5 × 10^6^ CFU were found after the inoculation of 1 × 10^5^ bacteria. In both wound infection models, no bacteria were extracted from uninfected control wounds injected with saline only. In the previous study [32], PVP foils, without Cipro and prepared with and without acetic acid, were also tested. Because they did not show any antimicrobial activity, they were not included in this study. The 20 h treatment with the Cipro-films (corresponding to about 500 µg antibiotic per wound) showed a complete eradication of both bacteria strains from the wound tissue.

### 3.4. Skin Immune Response

**Figure 4:**
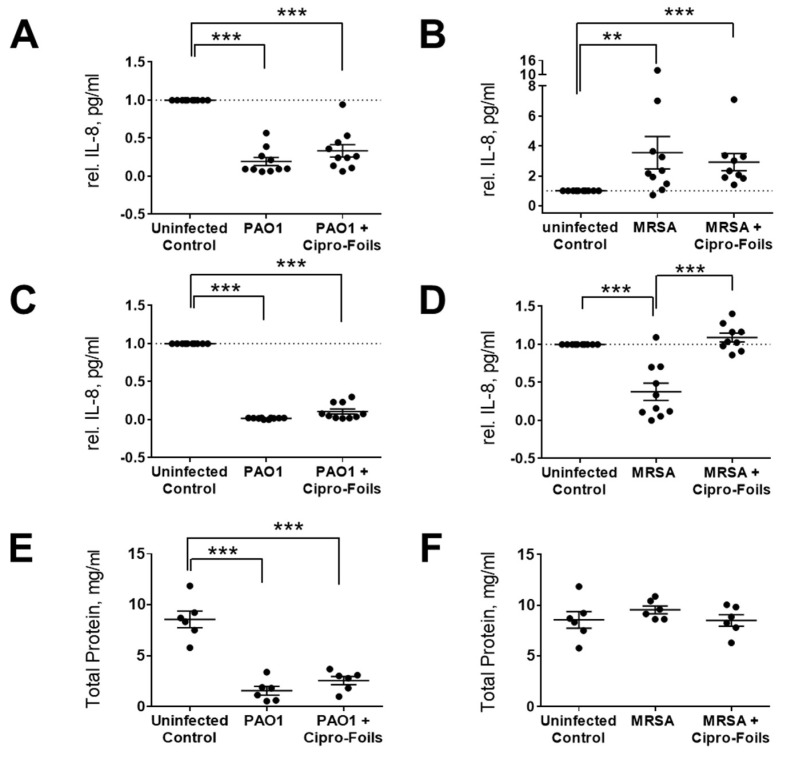
Effects of bacterial infection and its treatment in the ex vivo wound models. Levels of IL-8 in tissue extracts (**A**,**B**) and culture media (**C**,**D**) in ex vivo wound samples after wound infection with PAO1 (**A**,**C**) and MRSA (**B**,**D**) and with or without treatment with Cipro-foils. The amount of total protein in wound extracts of PAO1 (**E**) and MRSA (**F**) samples was also quantified. Data are normalized with respect to untreated controls and presented as the relative IL-8 concentration in wound extracts or medium. Dots represent the values for each sample. Arithmetic means and SEM are also shown. ** *p* < 0.01; *** *p* < 0.001.

To test the immunological response of skin in the ex vivo wound infection model with or without treatment with antimicrobial agents, the IL-8 released in wound tissue extracts as well as culture media was quantified using an ELISA assay (Figure 4).

The concentration of IL-8 in PAO1-infected wound samples was lower than in uninfected control wounds (Figure 3A,C). Even the amount of total protein in tissue extracts of PAO1-infected wounds was strongly reduced (Figure 3E). According to the manufacturer’s information, only proteins and peptides with a size ≥ 2500 Da can be detected with the Pierce 660 nm Protein Assay. Thus, knowing that PAO1 has strong proteolytic effects, these results suggest that PAO1 was able to digest the proteins of the ex vivo wound tissue, including IL-8. For this reason, the amounts of IL-8 were not normalized to the total extracted protein values but were normalized to the untreated controls. In general, IL-8 levels in PAO-1 infected wounds were always lower than those in the uninfected controls. Similarly, the IL-8 amounts in the culture media of infected wounds were always lower than that of the respective controls and always lower with respect to that of tissue extracts. Similar results were found also for infected wounds treated with the Cipro-foils.

Surprisingly, for the ex vivo infection model with MRSA, a completely different picture was found in relation to the IL-8 secretion (Figure 3B,D,F). The IL-8 levels detected in wound extracts after MRSA infection were on average 3.5 times higher than those of uninfected wounds. The average relative IL-8 value for the group treated with Cipro-foils was slightly lower than the infection group but three times higher than that of the control group (Figure 3B). Accordingly, the measured concentrations of total proteins in the extracts of MRSA-infected samples were similar for all groups: uninfected, infected, and treated. The values of the single samples were relatively spread, which was probably due to the differences in the donor-specific immune response (e.g., due to donor age or gender). This trend could not be confirmed by the analysis of IL-8 secretion in the culture medium. The presence of MRSA caused a reduction in the IL-8 released in the medium to an average value of 0.38 with respect to the control group. Nevertheless, this effect was counteracted by the treatment with the Cipro-foils with an average value of IL-8 similar to that of the control group.

**Figure 5:**
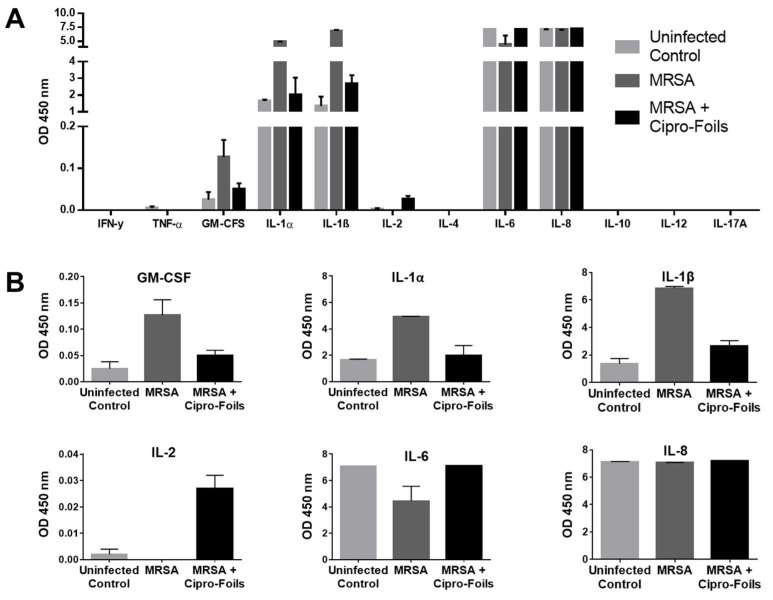
Analysis of inflammatory cytokines detected in the ex vivo MRSA infection model and effects of the topical treatment with Cipro-foils. The overview of the absorbance values for all the measured analytes (**A**) and the graphics with the adjusted scale for the detected analytes (**B**) are shown. Columns show arithmetic means and SD values of measured duplicates.

To detect further inflammatory cytokines stimulated by the infection with the MRSA strain and the possible effects of the antibiotic treatments, a multi-analyte ELISA was carried out (Figure 5). Extracts from three MRSA-infected samples and corresponding controls were pooled and analyzed.

No absorption, i.e., expression, of the cytokines IFN-γ, TNF-α, IL-4, IL-10, IL-12 and IL-17A could be detected. On the contrary, high levels of IL-6 and IL-8 were measured for both infected and non-infected wounds. In the case of IL-8, all samples reached the maximum OD value, and thus, it was not possible to show any differences between the groups. For IL-6, both uninfected and Cipro-foil treated samples reached the highest OD value, while the MRSA-infected wounds had a clearly lower OD, pointing to an inhibitory effect of the MRSA strain. IL-1α and IL-1-β were moderately expressed in uninfected wounds (OD ≅ 2), but their expression increased to higher levels (OD ≅ 5 and 7, respectively) in MRSA-infected wounds. Interestingly, the effects of MRSA infection on these cytokines could be counteracted by the treatment with the Cipro-foils. A similar effect was observed for GM-CSF even if the levels of this analyte were extremely low (OD = 0.04–0.16). Finally, a very low but slightly increased level of IL-2 could be detected in infected samples treated with the Cipro-foils. In contrast, there was no IL-2 signal for skin samples inoculated with MRSA or uninfected controls.

## 4. Discussion

Bacterial infections play a causal role in the development of chronic wounds. Two of the most common isolated pathogens are *Pseudomonas aeruginosa* and *Staphylococcus aureus*. Both possess a variety of virulence factors that allow them to invade the injured tissue, proliferate and evade the human immune response, especially in immune suppressed patients [32,33,34]. The development of new topical preventive and therapeutic procedures is important for the containment of chronic and nosocomial wound infections. On the other side, the selection of proper models that reflect host-specific characteristics and interactions with pathogens is required to test the efficacy of new materials. In previous investigations, a wound infection model with ex vivo human skin infected with PAO1 and incubated for 20 h in a humid chamber was characterized [25]. A biofilm-like accumulation of the predominantly aerobic strain on the wound surface was detected by electron microscopy. In this work, FISH was used to visualize both bacteria strains after a longer incubation time. This method allowed the direct detection of bacteria ribosomal RNA using fluorescence-labeled oligonucleotide probes. After 40 h of infection, biofilm-like structures were observed on the wound surface such as those found in the previous study after 20 h of incubation (Figure 1A–C). Fewer bacteria were found in the deeper dermis layers due to the lower oxygen content. MRSA bacteria were detected mainly in the wound edges but also in deeper dermis layers. Other than the PAO1 strain that formed a thicker biofilm covering almost the whole wound surface, the used MRSA strain formed less biofilm and with irregular clusters (Figure 1G–I).

To test the antimicrobial efficacy of the antibiotic topical treatment, a cryosection of wound infections treated with the Cipro-foils was also analyzed. The much lower fluorescent signal indicated that only a few, presumably dead, bacteria were present in the wound tissue. These results were confirmed by the CFU assay results (Figure 2). Cipro-foils were able to eradicate both bacterial infections 20 h after one single application. In our previous work, we demonstrated that the efficacy of Cipro-foils was related to the drug release kinetics. Specifically, delivery kinetics, performed in the same study using the ex vivo wound model, showed that PVP foils had a slower and more sustained drug delivery than the analogue PVP nanofiber mats [35]. This is probably the reason for the better antimicrobial activity of Cipro loaded on foils with respect to Cipro loaded on nanofiber mats. The results of this study confirm the previous findings using Gram-negative bacteria and demonstrate once again how a proper delivery and control of drug release can enhance its performance. In addition, they show that Cipro-foils are efficient also against a Gram-positive penicillin-resistant strain and are particularly relevant because they were obtained using human-derived, three-dimensional infection models.

Furthermore, the eventual effects of the infections and the topical antimicrobial treatment on skin inflammatory response, i.e., the expression of inflammatory markers, were investigated. The cytotoxic and proteolytic effects of PAO1 were evident. Compared to the previous study, the enzymatic effects toward IL-8 and skin proteins were even stronger. Whereas in the previous study, after 20 h of infection, the amount of IL-8 was similar to that of uninfected controls, in this study, after 40 h, the levels of IL-8 had diminished even more (Figure 3A,C). Even though the treatment with Cipro-foils eliminated all living PAO1 bacteria (Figure 2A), the proteolytic effects were not counteracted, as shown by the low concentration of total protein in the extracts (Figure 3E). These results show that bacterial proteases and toxins acted in the 20 h before the Cipro-foils were applied and that the effects of toxins continued despite the treatment. These findings are in accordance with previously reported data showing that cytokines are among the targets of bacterial proteases [36]. PAO1 has many extracellular proteases that are able to break down small peptides but also collagen fibers [37], which make up to 70% of the dermis components [38]. In addition, *S. aureus* strains possess collagen-degrading enzymes [37,39]. However, no extensive protein degradation was observed in the MRSA ex vivo model. The differences between PAO1 and MRSA infection models may be due to the fact that MRSA is non-motile and forms local colonies with restricted local nutrient resource [40]. This might limit the toxic effects of the *S. aureus* infection. Accordingly, in the tissue extracts of MRSA-infected samples, an increase in some inflammatory cytokines could be measured as reactions to the bacteria (Figure 3 and Figure 4). The amounts of IL-8, GM-CSF, IL-1α, and IL-1ß expression in infected samples were higher than in uninfected samples. IL-8 acts as a chemokine and is released by several cell types during infections. GM-CSF is a growth factor released by fibroblasts, endothelial cells, macrophages and other immune system cells also in response to pathogen-associated signals [41]. IL-1α and IL-1ß are mainly expressed by epithelial cells, monocytes and macrophages. They play a role in maintaining the skin barrier function [42]. In the case of acute barrier injuries, as during infections, there is an increase in the epidermal expression of these interleukins [43]. Interestingly, with the exemption of IL-8, the increase in these inflammatory cytokines could be reduced to the level of uninfected skin thanks to the treatment with Cipro-foils.

In the case of IL-6 and IL-2, a rather opposite effect was measured: the infected untreated samples had lower cytokine levels than the infected treated samples. Surprisingly, also in the culture medium, a lower amount of IL-8 was measured compared to uninfected and infected but treated wounds. These results may be explained by a selective inhibition of specific cell populations upon MRSA infection. In fact, it has already been reported that *S. aureus* inhibited IL-8 expression in human neutrophils [44]. Furthermore, Tajima et al. reported that beta-hemolysin reduced IL-8 production without cytotoxicity to endothelial cells [45]. Murphy et al. reported the same effect for V8 protease toward IL-6 [46]. Interestingly, this inhibitory or toxic effect was counteracted by Cipro-foils resulting in cytokine levels in medium comparable to those released by uninfected skin. Using the same ex vivo wound model and MRSA strain, Rosselle et al. reported similar results a thermo-sensitive cryogel dressing loaded with cefepime. They also achieved a complete eradication of the bacteria upon treatment. In addition, they observed an increase in IL-8, IL-1, and other cytokines’ mRNA upon skin infection along with a decrease upon treatment [47]. Whereas in our study lower levels of IL-6 protein were observed, the levels of IL-6 mRNA increased during infection in the study of Rosselle et al. However, this difference might be due to the detection method (PCR vs. ELISA) or the longer infection time (5 days vs. 2 days). Similar results were found also in an earlier study using human skin equivalents [48].

## 5. Conclusions

The infection of acute and chronic wounds is becoming a serious problem, especially in the hospital setting where patients are often immunosuppressed. The development of resistance and its spread is progressing rapidly. Therefore, the development of new delivery methods assuring a sustained and effective concentration of antimicrobials in the infected area is becoming urgent. This study shows that PVP foils loaded with the antibiotic drug ciprofloxacin using a co-solvent could achieve the complete eradication of biofilm-like colonies in the wound bed. Thus, we conclude that such transparent, easy to use, adhesive, adaptable foils are feasible and affordable dressings with both protection and antimicrobial functions for a wide range of applications.

## Data Availability

Not applicable.
